# Listeria monocytogenes Spontaneous Bacterial Peritonitis Associated With Dissemination and Fatal Meningoencephalitis in a COVID-19 Co-infected Cirrhotic Patient: A Case Report and Literature Review

**DOI:** 10.7759/cureus.105409

**Published:** 2026-03-17

**Authors:** Rodolfo Myronn De Melo Rodrigues, Luciana Vergara Ferraz de Souza, Rodrigo Furlan Silva Fabri, Vamsi K Kunam, Jeffrey Sherwood

**Affiliations:** 1 Internal Medicine, Texas Tech University Health Sciences Center El Paso, El Paso, USA; 2 Internal Medicine, University of Connecticut School of Medicine, Farmington, USA; 3 Interventional Radiology, The Hospitals of Providence Transmountain, El Paso, USA; 4 Infectious Disease, Texas Tech University Health Sciences Center El Paso, El Paso, USA

**Keywords:** co-infection, covid-19, listeria monocytogenes, meningoencephalitis, peritonitis

## Abstract

*Listeria monocytogenes* (LM) is a food-borne pathogen that can cause severe invasive infections, particularly in immunocompromised or elderly patients. Bacterial co-infections are frequently reported in COVID-19 patients, with both COVID-19-induced immune dysregulation and immunosuppressive therapies such as corticosteroids specifically creating risk for more unusual pathogens such as LM. We report a case of a 78-year-old woman with underlying cirrhosis due to non-alcoholic steatohepatitis who presented with fever and weakness, tested positive for COVID-19, and rapidly developed evidence of LM peritonitis with bacteremia and meningoencephalitis after dexamethasone initiation. Clinicians should maintain a high index of suspicion for listeriosis in acutely encephalopathic or septic patients with risk factors, especially during the COVID-19 era and in those receiving immunosuppressive therapy.

## Introduction

Since the onset of the COVID-19 pandemic, opportunistic bacterial and fungal co-infections have been increasingly described in association with severe acute respiratory syndrome coronavirus 2 (SARS-CoV-2) infection. In one review, it was noted that as many as 50% of patients who died from COVID-19 were reported to have secondary bacterial infections based on clinical and microbiological data, and that co-infection was associated with increased risk of shock, respiratory failure, prolonged hospitalization, and mortality [1]. Studies have suggested that the immune system may be suppressed in the early stage of COVID-19, with downregulation of several proteins important for an appropriate immune response [2].

*Listeria monocytogenes* (LM) is a facultative intracellular gram-positive bacillus capable of causing bacteremia and central nervous system (CNS) infection in elderly or immunocompromised individuals. Although relatively rare, with an annual incidence rate of 1 to 10 cases per million, human listeriosis can occur following consumption of contaminated food. The fatality rate can reach as high as 24 to 52% even with appropriate antibiotics [3]. Meningoencephalitis refers to inflammation of both the brain and its surrounding membranes and can rapidly lead to seizures, coma, and death. Patients with cirrhosis and diabetes represent recognized high-risk groups due to impaired host defenses. Cirrhosis impairs innate immune function and promotes bacterial translocation from the gastrointestinal tract, thereby predisposing patients to invasive infections.

LM meningoencephalitis in the setting of SARS-CoV-2 co-infection has been rarely described, with only isolated case reports published to date [4,5]. Percuoco et al. reported a case associated with multifocal abscess and rapid progression of neurologic symptoms in an immunocompetent patient whose initial symptoms were attributed to SARS-CoV-2 [4]. Seki et al. described a secondary case of LM infection in an elderly COVID-19 patient who required treatment with multiple antibiotics, including ampicillin, sulfamethoxazole/trimethoprim, and meropenem to achieve clinical improvement [5].

Recent reports and studies have specifically linked bacterial superinfections to dexamethasone therapy in COVID-19 patients and suggest that transient suppression of cellular immunity may contribute to invasive disease [6,7]. Similarly, LM is an extremely rare cause of spontaneous bacterial peritonitis (SBP). However, this syndrome has been described in case reports and series, including one case receiving prolonged glucocorticoid therapy for Crohn’s disease [8]. Emerging data suggest that LM-associated SBP may present with atypical ascitic fluid findings that do not meet conventional polymorphonuclear (PMN) thresholds, potentially delaying diagnosis and appropriate empiric antibiotic selection [9].

We present an unusual case of LM peritonitis associated with bacteremia and subsequent meningoencephalitis in a cirrhotic patient with COVID-19 co-infection whose clinical deterioration occurred shortly after corticosteroid initiation. This temporal association occurred in the setting of established baseline risk factors for invasive listeriosis, including cirrhosis and diabetes.

This case was presented as a poster presentation at the American College of Physicians National Conference in Boston, MA, April 2024.

## Case presentation

A 78-year-old woman with type 2 diabetes mellitus, class II obesity, and hepatic cirrhosis secondary to metabolic dysfunction-associated steatohepatitis (MASH), complicated by portal hypertension and ascites, presented with a two-day history of generalized weakness and fever.

The patient reported a normal diet that included commercially available pasteurized dairy products and denied consumption of unpasteurized dairy, deli meats, or raw foods. There were no reported community LM outbreaks in the area at the time. She also had poorly controlled type 2 diabetes mellitus, with a history of prior diabetic ketoacidosis (DKA). Before transfer to our unit, she presented with severe hyperglycemia (glucose >300 mg/dL) requiring an insulin infusion, likely worsened by recent dexamethasone therapy for COVID-19. After stabilization, she was transitioned to basal insulin with sliding-scale coverage, with glucose levels improving to 156-208 mg/dL.

On arrival, she was febrile (103 °F), mildly hypoxic with an O₂ saturation of 88% on room air, and otherwise hemodynamically stable without vasopressor requirement. Examination was notable for a distended but non-tender abdomen, bilateral lower-extremity edema, and intact cognition.

Laboratory data revealed mild lactic acidosis and peripheral leukocytosis. SARS-CoV-2 polymerase chain reaction (PCR) was positive. Blood cultures were obtained at the time of admission as part of the initial sepsis evaluation. CT chest angiography excluded pulmonary embolism, although ascites was confirmed on abdominal imaging. Her Model for End-Stage Liver Disease (MELD) score was 17. She was classified as Child-Pugh Class B (score 9) at presentation. She was started on intravenous remdesivir, dexamethasone, and empiric ceftriaxone due to concern for possible SBP.

Within 24 hours of admission, she developed acute encephalopathy, generalized seizures, and progressive hypoxia requiring intubation. Atrial fibrillation with rapid ventricular response emerged; however, she remained hemodynamically stable. Management focused on rate control and anticoagulation risk stratification. Given her CHA₂DS₂-VASc score of 4 (age ≥75 years, hypertension, and female sex), a heparin infusion was initiated following cardiology consultation, with close monitoring for bleeding in the setting of cirrhosis. An initial non-contrast head CT was unremarkable. Electroencephalography demonstrated moderate to severe encephalopathy without ongoing seizure activity.

Given worsening clinical status and concern for SBP, diagnostic paracentesis was performed. Ascitic fluid analysis revealed 247 nucleated cells/mm³ with 0% neutrophils (absolute polymorphonuclear count 0 cells/mm³), which did not meet the conventional ≥250 PMN/mm³ diagnostic threshold for SBP. However, the ascitic fluid culture subsequently grew LM. Initial admission blood cultures remained negative, but repeat blood cultures obtained later in the hospital course grew LM, confirming invasive bacteremia. On hospital day four, repeated non-contrast head CT imaging demonstrated a new small bilateral frontal subarachnoid hemorrhage, and anticoagulation was discontinued.

Intravenous ampicillin (12 g/day) was initiated as definitive therapy. Gentamicin was added for synergistic coverage. Therapeutic drug monitoring demonstrated a supratherapeutic gentamicin trough level of 3.9 µg/mL, accompanied by rising serum creatinine, raising concern for nephrotoxicity. Consequently, gentamicin was discontinued after approximately two days of adjunctive therapy.

**Figure 1:**
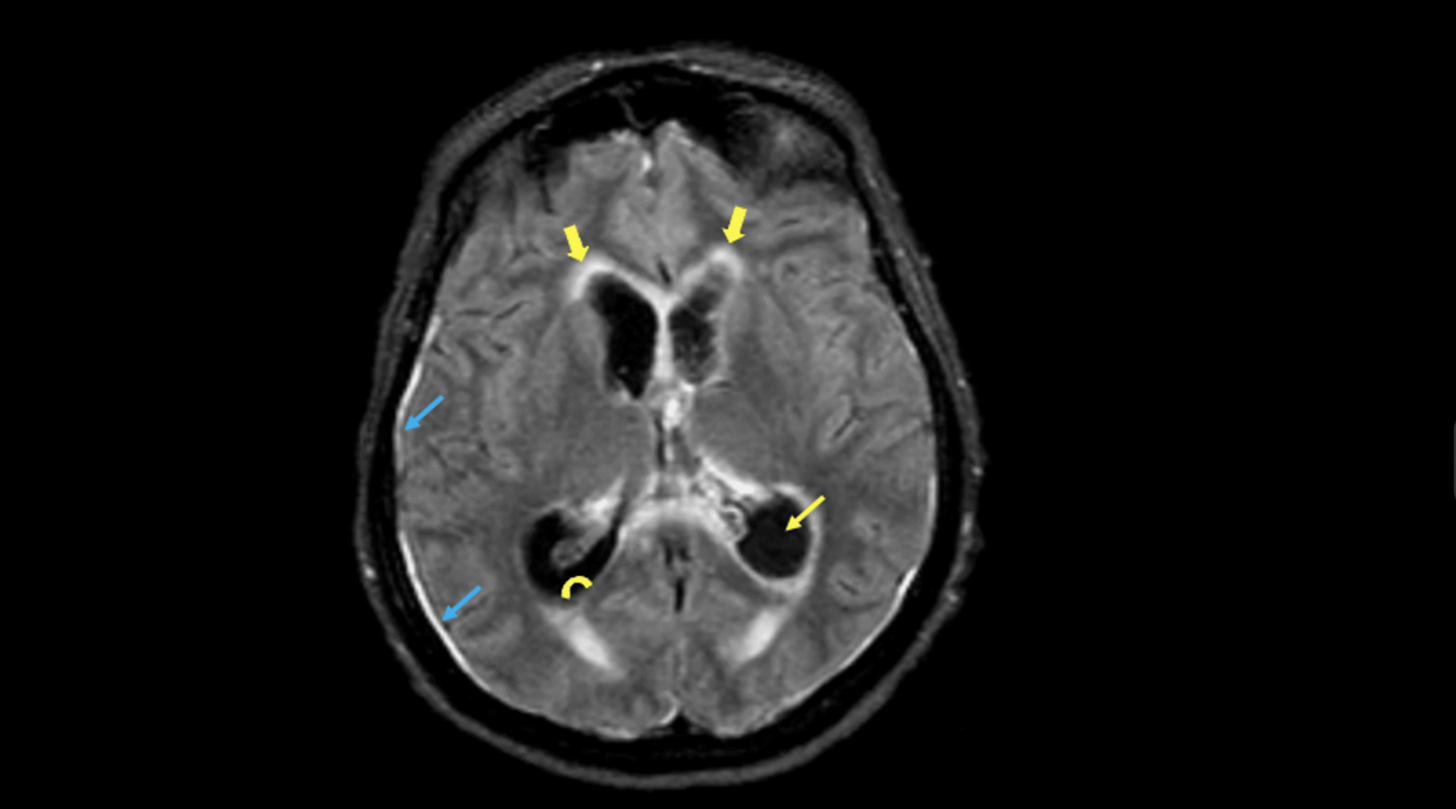
Imaging features of meningitis and ventriculitis on axial FLAIR MRI. Shows diffuse periventricular FLAIR signal (thick yellow arrows), hydrocephalus (thin yellow arrow), layering debris (curved yellow arrow), and meningeal hyperintense signal (blue arrows). FLAIR: fluid-attenuated inversion recovery.

Lumbar puncture revealed a cerebrospinal fluid (CSF) white blood cell count of 525 cells/µL and protein >600 mg/dL. CSF PCR was positive for LM, confirming meningoencephalitis, although CSF culture remained sterile. Axial fluid-attenuated inversion recovery (FLAIR) magnetic resonance imaging (MRI) of the brain demonstrated diffuse periventricular hyperintense signal, ventriculomegaly consistent with hydrocephalus, dependent layering debris within the ventricular system, and diffuse meningeal hyperintensity (Figure 1).

**Figure 2:**
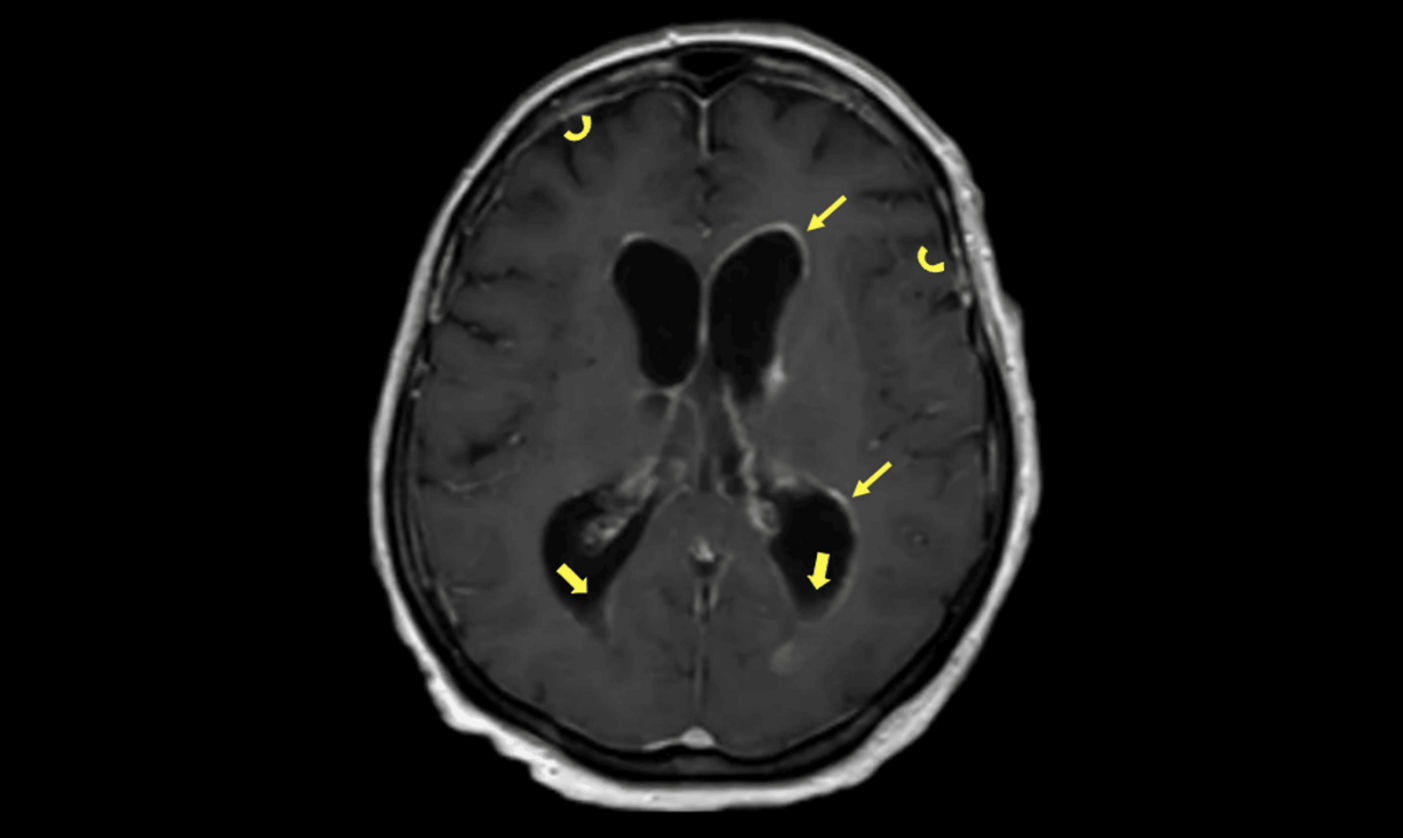
Post-contrast axial MRI of the brain. Demonstrates continued evidence of ventriculitis and meningitis: diffuse ependymal enhancement (thin yellow arrows), meningeal enhancement (curved yellow arrows), and layering debris ( thick yellow arrows).

Despite ongoing antibiotic therapy, neurological function continued to decline, and a repeat CSF PCR after 12 days of treatment was still positive. Repeat post-contrast axial MRI demonstrated diffuse ependymal enhancement lining the ventricular system, prominent leptomeningeal enhancement, and dependent intraventricular layering debris (Figure 2).

The patient remained comatose despite cessation of sedation. Given her poor prognosis, her family decided that palliative care was in her best interest, and she died a few days after extubation.

## Discussion

LM infections predominantly affect neonates, pregnant women, the elderly, and immunocompromised hosts, including those with cirrhosis or diabetes [10,11]. Invasive disease manifests as bacteremia or CNS infection, particularly rhombencephalitis and meningoencephalitis [12]. Cirrhosis itself predisposes to LM infection through impaired neutrophil function, reduced complement activity, and increased bacterial translocation [13]. Previous studies have demonstrated increased mortality from sepsis in cirrhotic patients regardless of the bacterial pathogen [14]. In a large prospective study from Taiwan, only 17% of 115 nonperinatal LM cases had underlying liver cirrhosis, making it a less common comorbidity compared to solid malignancy (47.1%) or steroid use (39.1%) [15]. Although LM septicemia has been reported in alcoholic cirrhosis as a sole risk factor, other cases have included additional immunosuppressive factors such as recent corticosteroid exposure or diabetes [16].

In our case, the patient had multiple established risk factors for invasive listeriosis, including cirrhosis and poorly controlled type 2 diabetes. Clinical deterioration occurred shortly after initiation of dexamethasone for COVID-19; however, this represents a temporal association rather than definitive causation. While corticosteroid exposure may have contributed to transient suppression of T-cell-mediated immunity, the presence of significant baseline comorbidities likely played a substantial role in disease susceptibility and progression.

The sequence of peritoneal fluid LM growth preceding bacteremia is uncommon, although LM peritonitis has been described in patients with ascites. In a nationwide French review of 208 LM-associated SBP cases, presenting symptoms were often non-specific. Notably, 109 (52%) patients died within six months of diagnosis, underscoring the high mortality associated with this infection in immunocompromised hosts. Importantly, 21 of 87 (24%) cases with available data had ascitic fluid polymorphonuclear (PMN) counts ≤250 cells/mm³ in the setting of inflammatory syndromes or bacteremia, prompting the authors to question the reliability of conventional diagnostic thresholds for LM-associated SBP [9].

Our patient’s ascitic fluid demonstrated 247 nucleated cells/mm³, with 0% neutrophils (absolute PMN count 0 cells/mm³), were well below the traditional ≥250 PMN/mm³ threshold for SBP despite a positive culture for LM. This case, therefore, reinforces an important clinical implication: ascitic fluid cultures should be obtained in high-risk cirrhotic patients even when PMN counts do not meet conventional diagnostic criteria, particularly when clinical suspicion persists.

Rapid progression to CNS involvement in this case underscores the pathogen’s neurotropic behavior and intracellular persistence. Despite appropriate high-dose ampicillin therapy, CSF PCR remained positive after 12 days of treatment, reflecting persistent infection. Adjunctive gentamicin was administered briefly; however, therapeutic drug monitoring demonstrated supratherapeutic trough levels, and the medication was discontinued due to concern for nephrotoxicity. Persistent CSF PCR positivity and ventriculitis on follow-up imaging underscore the therapeutic challenges of severe LM meningoencephalitis, particularly in cirrhotic hosts.

The diagnostic evaluation during neurological decline required consideration of a broad differential diagnosis, including metabolic encephalopathy (hepatic and hyperglycemia-related), seizure activity, cerebrovascular event, and infectious meningoencephalitis. Neuroimaging, electroencephalography, laboratory studies including ammonia levels, and ultimately CSF analysis guided the final diagnosis.

The COVID-19 milieu contributes additional immune dysregulation, lymphopenia, cytokine imbalance, and widespread corticosteroid exposure, which collectively may increase susceptibility to opportunistic infections [6,7]. Studies have demonstrated decreased expression of genes associated with adaptive immunity in severe COVID-19, particularly in T- and B-cell pathways and human leukocyte antigen (HLA) molecules [17]. Clinicians should therefore maintain vigilance for listeriosis in COVID-19 patients who develop acute encephalopathy or sepsis, especially in those with underlying cirrhosis, diabetes, or recent immunosuppressive therapy.

This report has limitations. As a single case, its findings are not generalizable. Genomic typing of the LM isolate was not performed, limiting epidemiologic characterization. Additionally, although deterioration followed corticosteroid initiation, causality cannot be definitively established given the patient’s substantial baseline risk factors.

## Conclusions

Invasive LM infection remains an important diagnostic consideration in elderly and immunocompromised patients presenting with sepsis or altered mental status. This case highlights the potential for atypical presentations of LM-associated SBP, including ascitic fluid findings that fall below conventional PMN thresholds despite culture-confirmed infection. Clinicians should consider obtaining ascitic fluid cultures in high-risk cirrhotic patients even when PMN counts do not meet standard diagnostic criteria, particularly when clinical suspicion persists.

The case also underscores the diagnostic complexity in patients with cirrhosis and COVID-19 co-infection, where overlapping metabolic, infectious, and neurologic processes may delay recognition of invasive disease. Although clinical deterioration occurred after corticosteroid initiation, this represents a temporal association rather than definitive causation in a patient with multiple established risk factors, including cirrhosis and diabetes.

Prompt recognition, early empiric ampicillin therapy when indicated, and close multidisciplinary management remain critical given the high mortality associated with CNS involvement in invasive listeriosis.
